# A critical review of autopsy findings in cases of suicidal hanging and the effect of a layered neck dissection

**DOI:** 10.1007/s00414-026-03917-8

**Published:** 2026-07-08

**Authors:** James Robert Lyness, Guy Rutty, Jane Rutty, Michael Biggs

**Affiliations:** 1 The State Pathologist’s Department, Belfast, UK; 2 East Midlands Forensic Pathology Unit, University of Leicester, Leicester, UK

**Keywords:** Hanging, Suicide, Neck Dissection, Hyoid fracture, Thyroid fracture, Neck muscle bruising

## Abstract

Suicidal hanging is a common case type referred to death investigation services throughout the world. It is associated with a large number of autopsy findings, but the presentation can vary significantly between cases, based on a multitude of factors related to the decedent, ligature and degree of suspension. There is also considerable variation in the reported incidence of autopsy findings within the published literature, some of which may be explained by different anthropometric characteristics of the included cases. However, study design and the thoroughness of the autopsy examination undertaken also undoubtedly influence results. This study therefore aimed to retrospectively review a series of autopsy reports from cases of suicidal hanging to assess not only the incidence of common findings identified during Coronial autopsies recently undertaken within a single region of the United Kingdom (UK), but also how the quality or thoroughness of the examination influenced their rate of detection. The results demonstrated considerable variation in the detection rates of common autopsy findings between individual consultant forensic pathologists and that the detection of neck muscle bruising (p = < 0.001), hyoid bone fractures (p = < 0.001) and thyroid cartilage fractures (*p* = 0.009) was greater when a layered neck dissection was undertaken. As a secondary objective, the range, incidence and impact of any natural disease and trauma additional to the ligature mark identified during the autopsy were assessed. Despite the array of natural disease and trauma recorded, none was considered critical to providing the underlying primary medical cause of death to the Coroner.

## Introduction

Hanging is a common cause of unnatural death referred to death investigation services throughout the world. It is a form of asphyxia caused by compression of the neck secondary to the gravitational drag of the weight of the body, either in full or partially [1]. Whilst the majority of cases are suicidal, accidental and homicidal deaths also occur, and it is important that a high-quality death investigation is completed. However, there is variation in the approach taken and extent of the investigation, including autopsy procedures [2]. Indeed, some authors suggest that a full traditional invasive autopsy, including a layered neck dissection, should be completed in all cases [3], whereas others propose that an external examination alone may be sufficient if the mechanism of injury and manner of death are clear [4]. Furthermore, the value of the information obtained during a traditional invasive autopsy and therefore the justification for the procedure have been questioned [2, 5].

A large number of external and internal autopsy findings associated with the diagnosis of hanging are recorded within the published literature. However, there is considerable variation in the reported incidence of many of these. Whilst a degree of variation may relate to anthropometric factors such as the age and body composition of the deceased, ligature characteristics, ligature and suspension point position on the neck and degree of suspension, some authors have concluded that the differences may be due to the effect of a prospective vs. retrospective study design [6–10]. Alternatively, others believe that it is due to the thoroughness of the autopsy examination undertaken [6, 7, 11–13] and the techniques utilised [7, 8, 10–14], arguably questioning the quality of the postmortem examination.

This study therefore sought to review autopsy reports from cases of suicidal hanging to assess not only the incidence of common findings identified during Coronial autopsies recently undertaken within a single region of the UK, but moreover how the quality or thoroughness of the examination influenced their rate of detection. The study also considered the incidence and range of natural disease and trauma additional to the ligature mark identified during the autopsy, as well as the impact such findings may have on the medical cause of death provided to the Coroner, to critically assess the additional information gathered during a traditional invasive autopsy in cases of suicidal hanging, a topic considered in only a minority of the published literature.

## Method

All Coronial autopsies in Northern Ireland (NI) are completed within The State Pathologist’s Department (SPD), Belfast. The SPD case management system was therefore used to identify all cases of suicidal hanging within the region. To satisfy sample size calculations and to include cases that had undergone a full traditional invasive autopsy, including microscopy of the major organs, the years 2016–2018 were selected. The inclusion criteria were therefore any Coronial autopsy undertaken within NI during 2016–2018 which had the word “hanging” within the cause of death; and the manner of death was suicide. Exclusion criteria were set as a body cavity, to include the head, neck, chest, abdomen and pelvis, was not examined; and/or a full autopsy report was not available.

All the examinations were completed in ‘real-world’ conditions for the sole reason of a Coronial investigation. Furthermore, unlike many regions within the UK, where non-suspicious autopsies, including the majority of hanging cases, are completed by non-forensically trained histopathologists, all the autopsies in this study were undertaken by consultant forensic pathologists, all of whom had passed postgraduate examinations in autopsy practice and were Fellows of the Royal College of Pathologists, UK (RCPath). However, whilst the autopsies were to the standards set by the RCPath during the selected study years [15, 16], there was no UK or regional standardised approach to the examination of suspected suicidal hanging cases. Indeed, the decision to complete a layered neck dissection or ancillary investigations, such as alcohol and toxicological analyses, was at the discretion of the pathologist.

The autopsy report provided to the Coroner in each case was reviewed by the main researcher. This comprised the standardised proforma used within SPD and by each of the pathologists during the study period, to include descriptions of the external and internal examinations, as well as information relating to the deceased and scene examination as provided to the pathologist by the police. Demographic and positive and negative findings relevant to hanging, as well as any natural disease or trauma, were entered into a spreadsheet in IBM SPSS statistics software version 28. No additional information sources were utilised during the study.

Potential findings within the neck, such as neck muscle bruising and laryngo-hyoid fractures, were considered key components of the examination of a suspected suicidal hanging. A layered anterior neck dissection, to include the sternocleidomastoid, omohyoid, sternohyoid, sternothyroid and thyrohyoid muscles [17], was considered an indicator of a high quality or thorough invasive autopsy, based on more recent hanging specific guidance from the RCPath published during 2023, after the selected study years [18]. The examinations of the hyoid bone and laryngeal cartilages were palpatory at the time of autopsy with no instrument augmentation. A Chi-squared test was used to compare the incidence of positive findings in the neck in relation to whether or not a neck dissection was undertaken. A cut-off *p*-value of 5% was selected. Findings such as carotid artery lesions like Amussat’s sign, intravertebral disc haemorrhage known as Simon’s sign, bowel wall haemorrhages, argent lines and other less frequently reported entities were not evaluated in this study.

## Results

During the three years selected for the study, 2016, 2017 and 2018, ‘hanging’ was included in the cause of death in 411 coronial autopsy cases completed within SPD. Following review of the autopsy reports no cases were excluded from the analysis. Of the study cases 410 were considered suicidal following the autopsy. The manner of death of the single remaining case was initially considered as unknown, due to the deceased having recently accessed internet websites relating to auto-erotic asphyxiation. However, there were no additional scene or autopsy findings to support this, and the Coroner ultimately concluded that the death was a suicide. None of the study cases were homicidal.

The traditional invasive autopsies were completed by 8 consultant forensic pathologists (Table 1). The variation in the number of cases undertaken was simply due to the time each pathologist was employed within SPD during the study period. Pathologists 1 & 2 were in fulltime posts during all three study years and therefore completed the highest number of cases, 124 and 121 respectively. Pathologist 7 was employed fulltime within the department for two of the study years, 2017 and 2018. The remainder of the consultant pathologists worked as locums for varying lengths of time within SPD during the study period.

**Table 1:** No. of invasive autopsies completed by each consultant forensic pathologist

Pathologist	No. of cases	% of Cases
1	124	30.2
2	121	29.4
3	42	10.2
4	46	11.2
5	8	1.9
6	7	1.7
7	61	14.8
8	2	0.5

Of the cases included in the study 403 (98.05%) were treated as routine Coronial examinations. The remaining 8 (1.9%) were classified as suspicious by the police prior to the invasive autopsy. These included 5 deaths within prison, a case of a suspected assailant having been found hanging after an alleged murder and a case where the decedent had been assaulted shortly before being found hanging. In the final case deemed suspicious the deceased’s partner had allegedly watched the individual hang themselves on closed circuit television cameras. However, following the investigation all these deaths were considered suicidal.

**Fig. 1:**
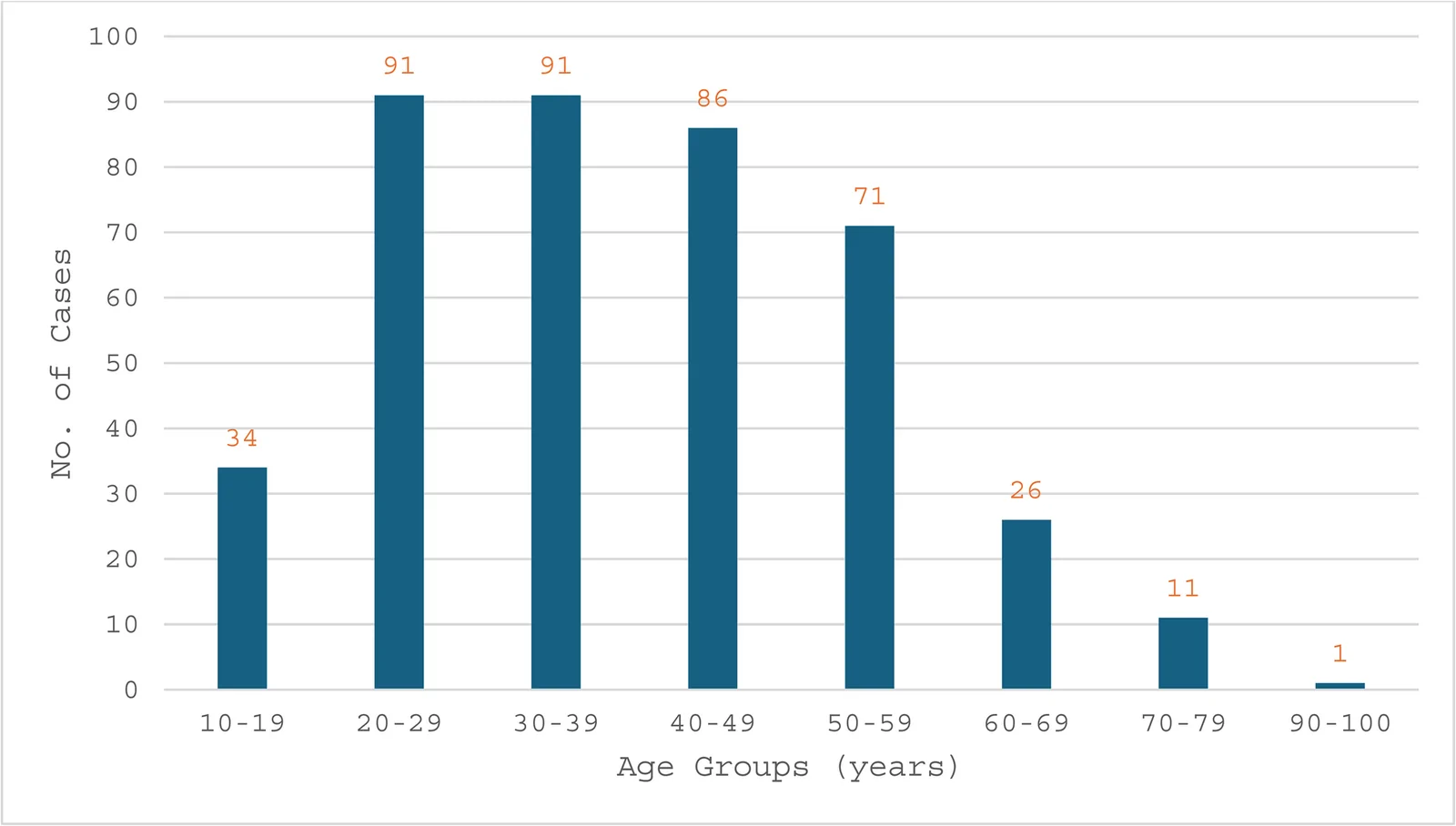
Bar chart demonstrating number of cases within age groups

Of the study cases 327 were male and 84 were female, equating to 79.6% and 20.4% respectively. The age of the decedents ranged from a minimum of 11.67 years to a maximum of 92.00 years, with an average of 39.68 years and standard deviation (SD) of 14.88. The age groups of 20–29 and 30–39 years were the largest, with 91 cases in each (Fig. 1).

### Findings specific to hanging

In 121 (29.4%) of the study cases the deceased was fully suspended when found hanging. In 209 cases (50.9%) the deceased was recorded as having been found partially suspended, as part of the body remained in contact with another surface. Of those remaining, 81 cases (19.7%), it was not known if the body was fully or partially suspended.

Conjunctival petechial haemorrhages were identified in 168 (40.9%) of the study cases. Crosstabulation showed that 40 of these were fully suspended decedents, 98 were partially suspended decedents and 30 were cases were the degree of suspension was not known, corresponding to 33.1%, 46.9% and 37.0% of each group respectively.

### Ligature

A wide range of ligatures were represented within the study cases. The most frequently utilised was a rope, accounting for 36.7% of cases (*n* = 151). Electrical cords, dressing gown belts and clothing belts were the next most common ligature types, accounting for 11.7%, 9.7% and 9.7% of cases respectively. In 7 cases (1.70%) material had been placed between the ligature and the underlying skin of the neck, including clothing (*n* = 4), a towel (*n* = 1), insulation (*n* = 1) and black elastic (*n* = 1), consistent with an attempt by the deceased to reduce any injury to the skin surface. Finally, in a single case the hands were described as clenched beneath the ligature.

In addition to a ligature around the neck, there were ligatures around the wrists and lower legs in 2 cases (0.49%) and around the wrists only in 1 case (0.24%). All of these additional ligatures could have been applied by the decedent and were interpreted as self-restraint. In a single case there was a piece of cloth within the mouth of the deceased, but this was not believed to have contributed to death and may represent an attempt by the deceased to reduce sound and/or pain. In another solitary case there was a plastic bag over the head of the deceased, albeit there was a hole within the bag in proximity of the mouth. The reporting pathologist concluded that the bag would not have obstructed breathing and may have simply been undertaken to mask the face. Therefore, despite these unusual findings all the above deaths were considered suicidal hangings.

### Ligature mark and suspension point

A ligature mark was identified in 405 cases (98.5%) during the autopsy. Therefore, in only 6 (1.5%) of the study cases no ligature mark was observed by the pathologist (Table 2). In 3 of these cases the decedent was partially suspended and in the remaining cases the degree of suspension was not known. Interestingly, the time from the individual last being seen alive and being found hanging was relatively short in 5 of the 6 cases (range 3-≤20 min; average 10.6 min). The ligature utilised was also composed of a soft material in 5 of the cases. Finally, apart from one case the individual survived for a period of time in hospital (range 3–20 days; average 8.2 days), potentially allowing any ligature mark to have undergone healing and to dissipate.

**Table 2:** Details of cases where no ligature mark was identified

Case No.	Degree of suspension	Duration of suspension	Ligature type	Suspension point identified	Pet Haems	Hyoid # NOS	Thyroid # NOS	Survival period
97	NK	∼ 5 min	Clothing	No	No	No	No	3 days
178	NK	≤ 20 min	Clothing	No	No	No	No	1 week
271	Partial	∼ 3 min	Shoelace	No	No	No	No	< 1 h
290	NK	NK	Dressing Gown Belt	No	No	No	No	20 days
346	Partial	< 10 min	Sheet/towel	No	No	No	No	1 week
403	Partial	< 15 min	Dressing Gown Belt	No	Yes	No	Yes	4 days

The ligature suspension point was clearly described by the pathologist in 394 cases (95.9%). The most common position was towards the back of the head/neck, 264 cases (64.2%). Only 12 (2.9%) of the study cases had an anterior/submental suspension point. Left and right sided suspension points recorded similar numbers, 60 (14.6%) and 58 (14.1%) respectively. In 17 cases (4.1%) a suspension point was not recorded by the pathologist.

### Additional neck injuries

Injuries of the neck in addition to the ligature mark were described in 33 (8.03%) of the study cases. Of these 24 (72.3%) were males and 9 (27.27%) females. The vast majority of the additional injuries recorded were considered minor non-specific bruises (11 cases) and abrasions (12 cases), and no significance was given to them by the reporting pathologist. However, in three cases the additional abrasion described was considered to have been most likely caused by slippage of the ligature during suspension, in a single case a band of abrasion was attributed to an underlying necklace and in another solitary case there was a second ligature mark caused by a cable tie which had compressed the neck but had not been involved in suspension. Self-harm incisions were identified on the neck in 3 (0.73%) of the study cases, one group of which appeared to be healing. Further non-specific healing abrasion was seen in a single case. Finally, needle puncture wounds were described on the neck in 2 cases, both attributed to medical treatment.

### Neck muscle bruising

Bruising of the anterior muscles of the neck, including the sternocleidomastoid, omohyoid, sternohyoid, sternothyroid and thyrohyoid muscles, was identified during the internal examination in 41 (10.0%) of the study cases. However, there was considerable variation in the detection rate between individual pathologists, from 0 to 28.57% (Table 3). There are undoubtedly multiple potential reasons for this, including low case numbers for some practitioners, pathologists 5, 6 & 8. In addition, whilst pathologists 2 & 4 removed the tongue and larynx during the evisceration and completed a palpatory examination of the hyoid bone and laryngeal cartilages, they did not routinely record having completed a layered anterior neck dissection, see below. Pathologist 6 was also the least experienced pathologist, having served less than three years as a consultant by the end of the study period.

**Table 3:** Detection rates for neck muscle bruising

Pathologist	Total No. of cases	No. of Cases with bruising	% of Cases
1	124	12	9.68
2	121	3	2.48
3	42	4	9.52
4	46	2	4.35
5	8	2	25
6	7	2	28.57
7	61	16	26.23
8	2	0	0

### Laryngo-hyoid Fractures

**Fig. 2:**
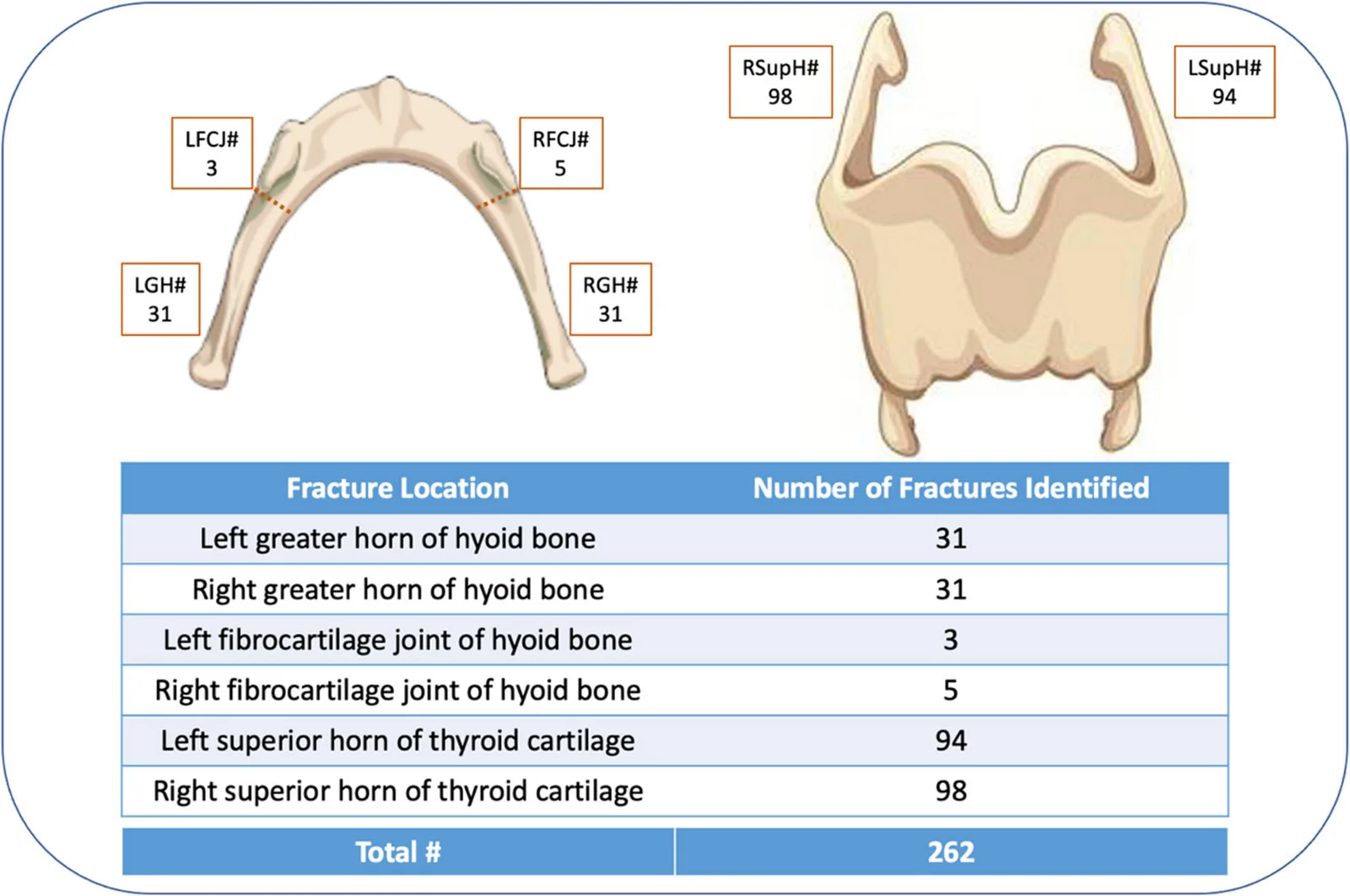
Frequency of laryngo-hyoid fractures identified by anatomical location.

A total of 70 fractures of the hyoid bone were identified within 58 (14.1%) of the study cases. Reassuringly, there were equal numbers of fractures detected within the greater horns, 31 for both left and right sides. Fewer fractures were detected within the fibrocartilage joints, 3 left-sided and 5 right-sided (Fig. 2).

Fractures of the thyroid cartilage were only described within the superior horns. A total of 192 individual fractures were identified within 131 (31.9%) cases. Again, there was minimal difference in the detection rates between left- and right-sided fractures, 94 and 98 respectively (Fig. 2).

No fractures of the cricoid cartilage were recorded by any of the pathologists.

Bruising/haemorrhage associated with fractures of the hyoid bone was identified in 22 cases, equating to 37.93% of those with hyoid fractures. A somewhat greater rate of bruising/haemorrhage was described in relation to fractures of the thyroid cartilage, 63.36%, corresponding to 83 cases.

Crosstabulations were also completed to assess the fracture detection rate of individual pathologists for both the hyoid bone and thyroid cartilage (Table 4). As before there was considerable variation in the detection rates, from 2.5 to 62.5% for hyoid fractures and 0–75% for thyroid fractures. However, again low case numbers for some of the pathologists may have caused a degree of bias within this data.

**Table 4:** Detection rates for laryngo-hyoid fractures

Pathologist	Total No. of cases	No. of cases with hyoid #	Detection Rate Hyoid #	No. of Cases with thyroid cartilage #	Detection rate thyroid cartilage #
1	124	23	18.5	50	40.3
2	121	3	2.5	26	21.5
3	42	14	33.3	22	52.4
4	46	6	13.0	10	21.7
5	8	5	62.5	6	75
6	7	1	14.3	2	28.6
7	61	5	8.2	15	24.6
8	2	1	50.0	0	0

### Neck Dissection

Of the 411 autopsy reports reviewed 244 (59.4%) referenced that a layered neck dissection was undertaken. In the remaining 167 (40.6%) cases no neck dissection was documented within the report, and the majority of these, 159 cases, were undertaken by two practitioners, pathologists 2 and 4 (Table 5). Whilst it remains possible that a layered neck dissection was completed and not recorded by the pathologist in the autopsy report, this was considered highly unlikely due to the use of the standardised proforma within SPD.

**Table 5:** Rate of layered neck dissection having been documented by pathologist

Pathologist	Total No. of cases	No. of cases with neck dissections	% of cases with neck dissection
1	124	118	95.2
2	121	4	3.3
3	42	42	100
4	46	4	8.7
5	8	8	100
6	7	6	85.7
7	61	60	98.4
8	2	2	100

Crosstabulations were subsequently completed to assess the detection of neck muscle bruising, hyoid bone fractures and thyroid cartilage fractures in relation to whether or not a neck dissection was undertaken (Table 6). In all three of these potential autopsy findings, the incidence of detection was greater in the cases where a layered neck dissection was completed, 39 (15.98%) vs. 2 (1.20%) for neck muscle bruising, 48 (19.67%) vs. 10 (6.02%) for hyoid bone fractures and 90 (36.89%) vs. 41 (24.70%) for thyroid cartilage fractures. Furthermore, chi-square tests demonstrated that these were statistically significant differences with *p-values* of < 0.001 for neck muscle bruising and hyoid bone fractures and 0.009 for thyroid cartilage fractures.

**Table 6:** Detection of neck findings in relation to whether a layered neck dissection was completed

Autopsy finding	Neck dissection completed		Neck dissection not completed	
	Detected	Not Detected	Detected	Not detected
Neck Muscle Bruising	39	205	2	165
Hyoid Bone Fracture	48	196	10	156
Thyroid Cartilage Fracture	90	154	41	125

### Natural disease

The natural disease recorded during the invasive autopsies was categorised based on the anatomical region it was detected in, i.e. the head, neck, chest and abdomen.

Some form of natural disease was detected within the head in 27 cases (6.57%). The most common finding was hypoxic ischaemic necrosis of the brain, in 3.89% of cases, and perhaps not unexpectedly in all 16 of these cases the deceased had been successfully resuscitated and had survived in hospital for a period of time. Of those remaining, the natural disease diagnosis was considered an incidental finding, including 2 cases of chronic cerebrovascular disease and 2 cases of cerebral atrophy, and single cases of meningioma, dural cyst, cerebellar cyst, capillary haemangioma and multiple sclerosis.

Natural disease was described in the neck in 22 (5.35%) of the study cases. All these diagnoses related to the thyroid gland, including 8 cases of chronic atrophy, 6 cases of benign nodular change and a single case of hypertrophy.

The chest was the most common anatomical region within which natural disease was recorded during invasive autopsy, with at least one diagnosis in 185 cases (45.01%). The entity most frequently recorded was coronary artery atheroma, in a total of 143 cases (34.79%). For most this was considered an incidental finding, but in two cases (0.49%), a recommendation was made to offer screening to family members for cardiovascular risk factors as the coronary artery disease detected was beyond what would typically be expected at the age of death, in decedents 32 and 24 years of age. Both recommendations were made by pathologist 1. No additional referrals for inherited conditions were made within the study cases.

Of note, pneumonia was diagnosed in 13 cases (3.16%). However, 9 of these were attributed to a terminal event secondary to prolonged survival in decedents with concomitant hypoxic ischaemic encephalopathy.

The final anatomical region considered was the abdomen and this included the pelvic cavity. At least one diagnosis of natural disease was identified in 88 (21.41%) of the study cases. Again, a range of entities were recorded, the most common including 21 cases of gallstones, 14 cases of alcoholic liver disease, 15 cases with minor gynaecological disease and 13 cases of prostate disease.

### Trauma

The internal trauma detected during the invasive autopsies was again reviewed within the four main anatomical regions, the head, neck, chest and abdomen. In 25 (6.08%) of the 411 cases there was evidence of trauma within the head. The vast majority of these, 22 cases (5.35%), comprised bruising of the undersurface of the scalp. In the final 3 cases (0.73%) there was evidence of an old brain injury. No skull fractures or acute intracranial haemorrhages were identified.

Excluding the ligature mark, other skin surface injury, neck muscle bruising or laryngo-hyoid fractures, additional trauma to the neck was recorded in 6 cases (1.46%). Five of these (1.22%) comprised fractures of the cervical spine, all of which were associated with haemorrhage, consistent with antemortem injuries. In four of these cases there was probably a drop from a height into the point of suspension. A fracture within the 5th cervical vertebra was the most commonly described, in 3 of the 5 cases. The cervical fracture was higher in the spine in one case, at the level of C2, and this involved almost complete decapitation. The single remaining case described haemorrhage within the paraspinal muscles of the cervical region, but no fracture was detected. In this final case the ligature had split, and the deceased was found on the floor beneath the point of suspension.

Trauma to the chest was recorded in 47 cases (11.44%). For the vast majority of these, 41 cases (9.98%), the traumatic injuries comprised anterior rib fractures, all of which were attributed to resuscitative chest compressions. Additional trauma was documented in 7 cases, including 4 (0.97%) with non-resuscitative rib fractures. Of these, 3 cases had associated bruising consistent with the rib fractures having occurred during life, one in a case with a suspected drop from a height into the point of suspension and another where the ligature had split, and the deceased was found on the floor beneath. In the third of these cases the pathologist concluded that the single rib fracture may have occurred during the hanging process. In the fourth case with non-resuscitative rib fractures there was no associated haemorrhage, but the decedent was found within an enclosed space, and these may have resulted from efforts to lower them from suspension. Thoracic spinal fractures were identified in 3 of the study cases (0.73%), one of which was a known recent antemortem crush fracture and another a healed fracture with callous formation. The third thoracic spinal fracture was not associated with haemorrhage and was considered a postmortem injury, possibly the result of manual handling.

In only 2 of the study cases (0.49%) was trauma within the abdominal cavity recorded during the invasive autopsy. Both of these cases were lacerations of the liver, and both appeared perimortem, with no significant associated intra-abdominal haemorrhage. The pathologists therefore concluded that these injuries were most likely caused by resuscitation attempts.

### Histology

Representative tissue samples from, at the very least, the major organs of the body were taken for histological examination by the pathologist in 399 (97.08%) of the study cases. Microscopic examination of the neck musculature or the laryngohyoid complex was not standard practice. The most common additional natural diseases identified by histology were hepatosteatosis in 76 cases (18.49%) and emphysematous change of the lungs in 28 cases (6.81%). Additional information pertaining to coronary artery disease was also identified microscopically, predominantly an increase in the assessment of the atheromatous narrowing rather than a new diagnosis.

A total of 16 (3.89%) of the decedents included in the study were successfully resuscitated and survived for a period of time in hospital. These cases had a range of terminal conditions, e.g. hypoxic ischaemic necrosis of the brain, myocardial necrosis and pneumonia +/- aspiration. However, in only 8 of these cases did the histological examination identify additional diagnoses not already made macroscopically, and two of these were incidental pre-existing natural disease.

The remaining cases comprised a wide array of microscopic diagnoses. However, none of these were considered significant to the cause of death.

Finally, in a small number of cases the microscopic examination was used to confirm antemortem diagnoses and injuries. Examples included confirmation of cancer (2 cases), exclusion of tumour (1 case), aging of a rib fracture (1 case) and confirmation/aging of a bruise of the labia (1 case).

### Alcohol Analysis

Only 16 cases (3.89%) included within the study did not have a sample of blood submitted for alcohol analysis. In 9 of these cases the decedent survived for a period in hospital and a relevant blood sample from the time of their admission was not available. Of the 395 cases (96.11%) where a sample of blood was submitted for alcohol analysis the average alcohol concentration was 67.46 milligrams per 100 ml., with SD of 92.85 and range of 0 to 423.00 milligrams per 100 ml.

Urine samples were available in fewer cases, 305 in total (74.21%). Of these the average alcohol concentration was 91.40 milligrams per 100 ml., with SD of 117.63 and a range of 0 to 386.00 milligrams per 100 ml.

### Toxicological analysis

A toxicological analysis was requested in 137 (33.33%) of the study cases. A range of common pharmaceutical medications and drugs of abuse were identified. However, of relevance to this study there was variation in the rate of ordering toxicology between the pathologists, 5.8–100% of their caseload (Table 7). In addition, tablets and/or tablet residue were identified within the stomach in 5 cases (1.22%).

**Table 7:** Frequency of toxicological analysis for individual pathologists

Pathologist	Total no. of cases	No. of cases with toxicology	% of Cases
1	124	48	38.7
2	121	7	5.8
3	42	24	57.1
4	46	17	37.0
5	8	8	100
6	7	7	100
7	61	25	41.0
8	2	1	50.0

## Discussion

Suicidal hangings are a common cause of unnatural death referred to death investigation services. A wide range of postmortem features has been historically associated with the diagnosis, including a ligature mark on the neck, inferior extremity hypostasis/lividity, pallor or less often congestion of the face, petechial haemorrhages, hyoid-laryngeal fractures, fractures of the cervical vertebrae and neck muscle haemorrhage/bruising. However, the variability of such findings across cases means there are no absolute pathognomonic features to make the diagnosis of hanging and increasingly the significance and validity of some findings are being questioned [19, 20]. It is therefore imperative that high quality investigations are undertaken and the evidence gathered critically assessed towards providing an accurate cause and manner of death [2].

Similar to many previous manuscripts [6–8, 10, 11, 14, 21–56] this study revealed that suicidal hanging is considerably more common in males, 79.6%. There was also an increased incidence of petechial haemorrhages within the partially suspended decedents compared to those fully suspended, 46.9% vs. 33.1%, which is similar to the study of Clément et al. [57]. The ligatures utilised largely reflected objects easily available in UK, such as a rope (36.7%), belt (19.4%) and electrical cord (11.7%). Furthermore, a ligature mark was nearly always visible to the examining pathologist, 98.5% of cases, its absence associated with partial suspension, a short suspension time, a soft ligature and/or a survival period. Similarly, a suspension point was described in the vast majority of cases, 95.9%, and it was most commonly located towards the back of the head (64.2%), i.e. typical hanging, in keeping with much of the published literature [7, 21, 24, 30, 31, 33, 35, 36, 41, 49, 52, 58].

As previously discussed, there is considerable variation in the incidence of common autopsy findings associated with suicidal hanging across the literature. Some authors cited an increased incidence in prospective studies compared to retrospective studies [6–10], whereas others considered it secondary to the thoroughness of the autopsy examination [6, 7, 11–13] and the techniques utilised [7, 8, 10–14]. The results of this retrospective study, including neck muscle bruising in 10% of cases, hyoid bone fractures in 14.1%, thyroid cartilage fractures in 31.9% and no cricoid cartilage fractures, have been outlined above and can add further data to the variation between manuscripts. However, perhaps more interesting was the variation in detection rates of such features, generally considered key findings in cases of suicidal hanging, between consultant forensic pathologists within a single department as identified in this study. Indeed, to our knowledge this has not been considered elsewhere within the literature. It is of course acknowledged that each hanging case represents a complex biomechanical event, and the presence or absence of autopsy findings will depend on multiple factors, such as the age and body composition of the deceased, ligature characteristics, ligature and suspension point position on the neck and degree of suspension. Therefore, the low case numbers undertaken by 3 of the 8 pathologists may have caused a degree of bias within their individual data. However, when detection rates were compared for the entire group of pathologists against whether a layered neck dissection was undertaken there was a statistically significant decrease in the incidence of neck muscle bruising and hyoid bone and thyroid cartilage fractures in the absence of this procedure, a finding not demonstrated elsewhere within the reviewed literature. Of course, these results could also be explained by the diagnostic diligence of individual pathologists, particularly as the majority of the examinations without a neck dissection having been undertaken were completed by 2 practitioners. However, regardless, the results reinforce the view that the thoroughness of the autopsy and techniques utilised will affect the detection rate of pertinent findings in cases of suicidal hanging. Furthermore, the variation in detection rates and quality of examination, as clearly demonstrated by this study, could be reduced by the standardisation of medico-legal autopsy procedures, as outlined by professional bodies [18, 59].

An array of natural disease was detected during the invasive autopsies in this study, an area not considered in detail elsewhere within the literature. The highest incidence of natural disease was detected within the chest (45.01%), followed by the abdomen (21.41%), head (6.57%) and neck (5.35%). In a minority of cases, 16 (3.89%), at least one such diagnosis was included within the cause of death formulation, including hypoxic ischaemic necrosis of the brain 16 cases (3.89%) and concomitant pneumonia 9 cases (2.19%). Both diagnoses were considered terminal events in decedents who had survived for a period of time following hanging. Therefore, none of the natural disease diagnoses made during the autopsies were considered critical to providing the underlying primary medical cause of death to the Coroner. Proponents of invasive autopsy may suggest that some natural diseases, such as hypothyroidism, could be associated with depression, and this in turn could increase the risk of suicidal ideation. However, despite some form of thyroid disease having been identified in 5.35% of the cases included in this study, no such connection was made by any of the pathologists. Alternatively, invasive autopsy may identify natural disease that could have consequences for the living, e.g. inherited conditions. Indeed, in 2 cases (0.49%) included in this study such disease was detected, both severe coronary artery disease in a young person and both referrals were made by the same pathologist. However, whilst it is difficult to argue that such findings could be of a high value to the families involved, this is not inquiry legally expected from the Coroner in the UK.

Additional trauma identified during the autopsy in the study cases was also limited, and therefore if injuries are identified they require explanation and further investigation to exclude, as far as possible, third party involvement in the death. Rib fractures secondary to resuscitation were the most common entity, 41 cases (9.98%). Furthermore, whilst bruising of the undersurface of the scalp was recorded in 22 cases (5.35%), none was associated with skull fracture or an acute brain injury, albeit the main author has dealt with such cases in the past. In 3 cases (0.73%) there was evidence of an old brain injury, a finding that could potentially be associated with behavioural changes and an increased risk of suicide. However, any such extrapolation by a pathologist would, in our opinion, be speculative, and would be more appropriately diagnosed on antemortem clinical evidence. Of interest specific to hanging cases, fractures of the cervical spine were identified in 5 cases (1.22%), and in 4 of these the deceased probably dropped from a height into suspension, a finding consistent with the published medical literature [52].

As expected for the years selected for this study, almost all of the cases (97.08%) had some form of histological examination. However, the yield of new diagnoses generated was limited with the majority of findings, such as mild hepatosteatosis and emphysema, considered incidental. In a small number of cases the histological examination was used to confirm antemortem diagnoses and injuries, such as confirmation/exclusion of cancer, and aging of relevant injuries. Therefore, whilst histology does not produce additional information in the vast majority of cases, it can assist the pathologist/Coroner in a small number of suicidal hangings, a finding similar to the conclusions of Tse et al. [60]. Such cases should be identified following careful consideration by the death investigators.

The final stage of the traditional invasive autopsy considered was the use of ancillary investigations. Alcohol and toxicological analyses were completed in 96.10% and 33.33% of cases included in the study respectively. Whilst the results did not alter the cause of death provided by the pathologist, the presence of alcohol and/or drugs may assist the Coroner in establishing the circumstances leading to the death. However, similar to some of the pathological findings there was considerable variation in the practice of ordering toxicology between the pathologists, 5.8–57.1% of their case load. This inconsistency of practice within a single department and region again highlights the significant variation in approach that is taken in suspected suicidal hangings. These decisions are arguably based on the individual pathologist’s views and risk appetite, but also demonstrate a mismatch of quality of investigation and/or use of resources. Again, such findings support the need to standardise the approach to medico-legal autopsies, to ensure consistency and provide not only quality, but relevant information to the investigating authority.

### Summary

Our study has demonstrated considerable variation in the detection rates of common autopsy findings in cases of suicidal hanging between consultant forensic pathologists within a single institution within the UK. In particular, the study is the first to show that detection rates of pertinent findings, such as neck muscle bruising and hyoid bone and thyroid cartilage fractures, increase significantly when a layered neck dissection was undertaken, a procedure considered an indicator of a high quality or thorough invasive autopsy in this case type. The results therefore demonstrate the potential for variation in autopsy technique to effect findings and support the requirement to standardise medico-legal autopsy procedures and protocols, either locally or by national/international professional bodies, to ensure quality, consistency and the evidentiary value of medico-legal examinations in cases of suspected suicidal hanging. Finally, despite the array of natural disease and trauma having been detected during the autopsy examinations, none was considered critical to providing the underlying medical cause of death to the Coroner.

## Data Availability

The datasets generated during the current study are not publicly available as the information is the legal property of the Coroner for Northern Ireland.
